# Polymorphisms of the *prion protein* gene (*PRNP*) in Alaskan moose (*Alces alces gigas*)

**DOI:** 10.1111/j.1365-2052.2006.01466.x

**Published:** 2006-08

**Authors:** H J Huson, G M Happ

**Affiliations:** Institute of Arctic Biology, University of Alaska Fairbanks Fairbanks, AK 99775-7000, USA

## Source/description

The *prion protein* (*PRNP*) gene of mammals encodes a prion protein (PrP), which is expressed in many tissues including the brain. Misfolded PrP conformers are responsible for neurodegenerative diseases known as spongiform encephalopathies. Transmissible spongiform encephalopathies (TSEs) include bovine spongiform encephalopathy, ovine scrapie, human Creuzfeldt–Jakob disease and chronic wasting disease (CWD)^1^,^2^ in mule deer (*Odocoileus hemionus*), white-tailed deer (*Odocoileus virginianus*) and Rocky Mountain elk (*Cervus elaphus*). First found in Colorado, CWD has now been identified in the eastern USA, as far south as New Mexico and as far north as west-central Canada.^3^

Polymorphisms of *PRNP* appear to be linked to susceptibility to TSE in numerous species including free-ranging white-tailed deer^4^ and mule deer.^5^ In mule deer, the *SS* genotype at residue 225 is associated with a higher incidence of CWD.^5^ Differences in PrP amino acid sequence are believed to be species barriers to disease transmission.^6^ However, Wyoming moose sequences that were previously deposited in GenBank (AY225484 and AY225485) are similar to the sequence of *Odocoileus*. CWD has not been observed in Rocky Mountain moose (*Alces alces shirasi*) or in caribou at higher latitudes (*Rangifer tarandus*), yet both species overlap the geographical range of *Odocoileus* species. We report here the *PRNP* sequences for 44 Alaskan moose (*Alces alces gigas*).

## Polymerase chain reaction conditions and sequence analysis

Genomic DNA was purified from blood samples of 44 moose (*Alces alces gigas*) that were sampled from eight locations across Alaska. DNA purification protocols, primers, amplification conditions and sequence analysis methods are provided in Appendix S1.

## Polymorphisms

Two unique sequences (i.e. alleles) were found in the sequences of 44 individual moose (DQ154297 and DQ154298); these differed only at codon 209. The allele encoding methionine was present with a frequency of 0.45, and the allele encoding isoleucine was present with a frequency of 0.55. The diploid genotypes did not depart significantly from Hardy–Weinberg predictions (*χ*^2^ = 0.4, *P* < 0.01).

## Comments

The conservation of amino acid sequences in the PrP of moose, caribou and deer is striking (Table 1) and consistent with the fact that all three genera are in the subfamily Capreolinae. In comparison with caribou, Alaskan moose samples show six synonymous substitutions (bases 195, 231, 324, 360, 384 and 674), presumably reflecting purifying selection for the unique conformation of the globular N-terminal domain of cervid prions.^7^ CWD has been transmitted to moose by an oral route in an experimental laboratory setting.^8^ Genetic similarities, susceptibility in the laboratory setting and overlapping geographical ranges suggest the lack of a barrier to the transmission of prion disease from mule and white-tailed deer to moose. The absence of reports of CWD transmission to moose in natural settings may reflect ecological or epidemiological factors. Moose tend to be more solitary than deer of the genus *Odocoileus*, and dense social aggregations might be prerequisites of CWD epizootic outbreaks in cervids.

**Table 1:** A comparison of differences among the *prion protein* alleles of moose (DQ154297 and DQ154298), caribou (DQ154293) and mule deer (AY228473).

	Nucleotide position												
	
	4	195	231	324	360	384	385	413	438	505	618	627	674
Moose	G	A	G	C	A	T	G	G	T	G	T	G/T	T
Caribou	G/A	G	T	A	C	C	G/A	G/A	C/T	G/A	T	G	C
Mule deer	A	A	T	A	A	C	G	G	T	G	C	G	C

	Codon												
	
	2	65	77	108	120	128	129	138	146	169	206	209	225
Moose	V	G	G	P	A	L	G	S	N	V	I	M/I	S
Caribou	V/M	G	G	P	A	L	G/S	S/N	N	V/M	I	M	S
Mule deer	V	G	G	P	A	L	G	S	N	V	I	M	S

Unlisted bases and amino acids were identical.

## Supplementary Material

The following supplementary material is available as part of the online article from http://www.blackwell-synergy.com:

Appendix S1 Table of differences among prion alleles, Materials and Methods.
