# Supplementary material for: Polymorphisms of the prion protein gene (PRNP) in Alaskan moose (Alces alces gigas)
Source: Anim Genet. 2006 Aug;37(4):425–6. doi: 10.1111/j.1365-2052.2006.01466.x (PMC1592321; doi:10.1111/j.1365-2052.2006.01466.x)
Supplement: Appendix S1 — Table of differences among prion alleles, Materials and Methods. [file age0037-0425-AppendixS1.doc]

# Supplemental Materials

*Biological materials* -Whole blood samples were obtained from Alaskan moose (Alces alces gigas). Samples were from eight different interior and southwest Alaska sites (Figure S1). Genomic DNA was extracted using the QIAGEN QIAamp DNA Blood Mini Kit. QIAGEN protease was utilized for cell lysis. The genomic DNA was eluted with deionized water. Forty-four Alaska moose were successfully sequenced.

*Polymerase chain reaction* - PCR reactions were performed with 50-100 ng DNA in a 50-μl volume containing 1x PCR buffer as supplied by Qiagen (with no extra magnesium), 0.2 mM of each dNTP, 0.5 pmol/μl of both forward and reverse primers, and 0.025 U/μl Qiagen HotStarTaq Polymerase. A modified forward primer, Ce19v2- CTTTATTTTGCAGATAAGTC and reverse primer of Ce778- AGAAGATAATGAAAACAGGAAG were utilized (O’Rourke *et al.*, *J Gen Virol*. **80**, 2765-9, 1999*)* An MJ Research PTC-225 Peltier Thermal Cycler was used with the following settings: 95°C for 15min; then 40 cycles of 94°C for 30 sec, 51.5°C for 30 sec and 72°C for 1 min; with a final extension of 72°C for 10 min.

## PCR product purification and cycle sequencing - PCR purification was performed on all samples using the QIAGEN QIAquick PCR Purification Kit. All samples were eluted with 50 μl deionized water. Cycle sequencing was performed using Big Dye Terminator Version 3 (Applied Biosystems). Sequence purification was performed with sephadex G-50 DNA grade, and the samples were sequenced on an Applied Biosystems 3100 Genetic Analyzer.

## Sequence analysis - Raw sequence data were analyzed using the Sequencher (GeneCodes Inc.) and BioEdit ([*www.mbio.ncsu.edu/BioEdit/bioedit.html*](http://www.mbio.ncsu.edu/BioEdit/bioedit.html)) programs to yield 771-bp sequences of the prion protein.
