# Supplementary material for: Polymorphisms of the prion protein gene (PRNP) in Alaskan moose (Alces alces gigas)
Source: Anim Genet. 2006 Aug;37(4):425–6. doi: 10.1111/j.1365-2052.2006.01466.x (PMC1592321; doi:10.1111/j.1365-2052.2006.01466.x)
Supplement: Appendix S1 — Table of differences among prion alleles, Materials and Methods. [file age0037-0425-FigS1.doc]

*Sleetmute*

*McGrath*

*Lake Clark*

*Togiak NWR*

*Aniak*

*Fairbanks*

*Upper Birch Creek*

*Preacher Creek*

**Supplementary Figure 1** Locations of sites where moose were sampled in Alaska.
